# *Megasphaera* in the gut microbiome and cancer: from *Megasphaera elsdenii* dysbiosis to *Megasphaera* sp. XA511 in tumor microenvironments

**DOI:** 10.3389/fcimb.2026.1766220

**Published:** 2026-03-27

**Authors:** Rohan Kubba, Sameer Kejriwal, Jacob Razzouk, J. Robert Evans

**Affiliations:** 1 California Northstate University College of Medicine, Elk Grove, United States; 2 Ronald Reagan University of California Los Angeles (UCLA) Medical Center, Los Angeles, United States; 3 Loma Linda University School of Medicine, Loma Linda, United States; 4 University of California Riverside School of Medicine, Riverside, United States

**Keywords:** dysbiosis, gut microbiome, gut-lung axis, cancer, lung microbiome, Megasphaera, niche multiplicity, SCFAs

## Abstract

Growing evidence suggests that the gut microbiome and specific gut microbes influence carcinogenesis both within the gastrointestinal tract and in distant organs through immune, metabolic, and inflammatory pathways. Megasphaera elsdenii, a gram−negative-staining, strictly anaerobic member of the Veillonellaceae family, has been implicated in disruption of colonic epithelial homeostasis and may exert systemic effects beyond the intestine. While much attention has focused on the gut–brain axis, this mini−review synthesizes current evidence linking intestinal dysbiosis, microbial metabolite signaling, and immune crosstalk along the gut–lung axis. By integrating findings from studies on microbial translocation, mucosal immunity, and metabolite−mediated inflammation, we present a hypothesis−generating model in which M. elsdenii–driven gut dysbiosis may shape lung cancer pathogenesis through short−chain fatty acid–dependent immunometabolic signaling and hypothesized lymphatic and outer membrane vesicle–mediated pathways, recognizing that existing lung data derive solely from non−causal, genus−level 16S rRNA surveys. We further distinguish viable colonization from detection of immunogenic DNA and vesicular debris in distal tissues and discuss the context−dependent roles of the genus, contrasting the systemic pathogenicity of M. elsdenii in the gut–lung axis with the divergent, protective metabolic profile of a distinct gut−derived strain, Megasphaera sp. XA511, in pancreatic tumor microenvironments. This framework highlights Megasphaera as an understudied but potentially actionable modulator of cancer immunobiology.

## 1 Introduction

*Megasphaera elsdenii* is a prevalent gut microbe found in ruminants associated with lactate management (Cabral and Weimer, 2024). In humans, *M. elsdenii* overgrowth is implicated in bacterial vaginosis via dendritic cell upregulation of CD80/83/86 (Van Teijlingen et al., 2020) and contributes significantly to gas production during carbohydrate fermentation (Mutuyemungu et al., 2023). It is also opportunistically pathogenic, evidenced by a rare case of endocarditis in a patient with a ventricular septal defect (Brancaccio and Legendre, 1979).

*M. elsdenii* has garnered attention for its dualistic behavior: commensal in metabolic balance yet potentially pathogenic in dysbiosis. Traditionally known for fermenting lactate into short-chain fatty acids (SCFAs) such as acetate, propionate, and butyrate, *M. elsdenii* maintains colonic energy balance (Chowdhury et al., 2015). SCFAs affect not only colonocytes but are also detectable in trace amounts in the bloodstream, capable of affecting cells in bone marrow hematopoiesis, including lymphoblasts (Thiruvengadam et al., 2023). *In vitro* assays have suggested that SCFAs upregulate mTOR activity and inhibit histone deacetylase I thereby leading to improvements in anti-tumor immunity (Luu et al., 2021). A 2013 study also found that some strains of *M. elsdenii* possess enzymes that produce essential amino acids such as lysine via the diaminopimelate pathway, as well as B vitamins including B12 (Shetty et al., 2013). However, its overgrowth in the gut has been associated with disruptions in epithelial junctions, increases in ROS, and activation of proinflammatory signaling (Fellows et al., 2024). Other *Megasphaera* species are oral commensals associated with periodontal disease when abundant (Nallabelli et al., 2016). *M. elsdenii*, as a non-motile, non-spore forming cocci (Srinivasan et al., 2021), influences sites inside and outside of the GI tract via both metabolic and immune routes.

Lung cancer remains the leading cause of cancer death worldwide (WHO, 2023). The lung microbiome has garnered attention as a prognostic biomarker, challenging the sterile lung dogma. A recent study by (Li et al., 2024) emphasized that the lung hosts a resident microbiome with significant influence from gut-derived signals. Two papers (Eladham et al., 2024; Zhao et al., 2025) further corroborate this model, emphasizing a bidirectional microbiota–immune axis, spanning gut and lung that communicates through cytokine gradients, lymphatic trafficking, and microbial metabolites, including short-chain fatty acids (SCFAs), lipopolysaccharides, and secondary bile acids.

**Figure 1:**
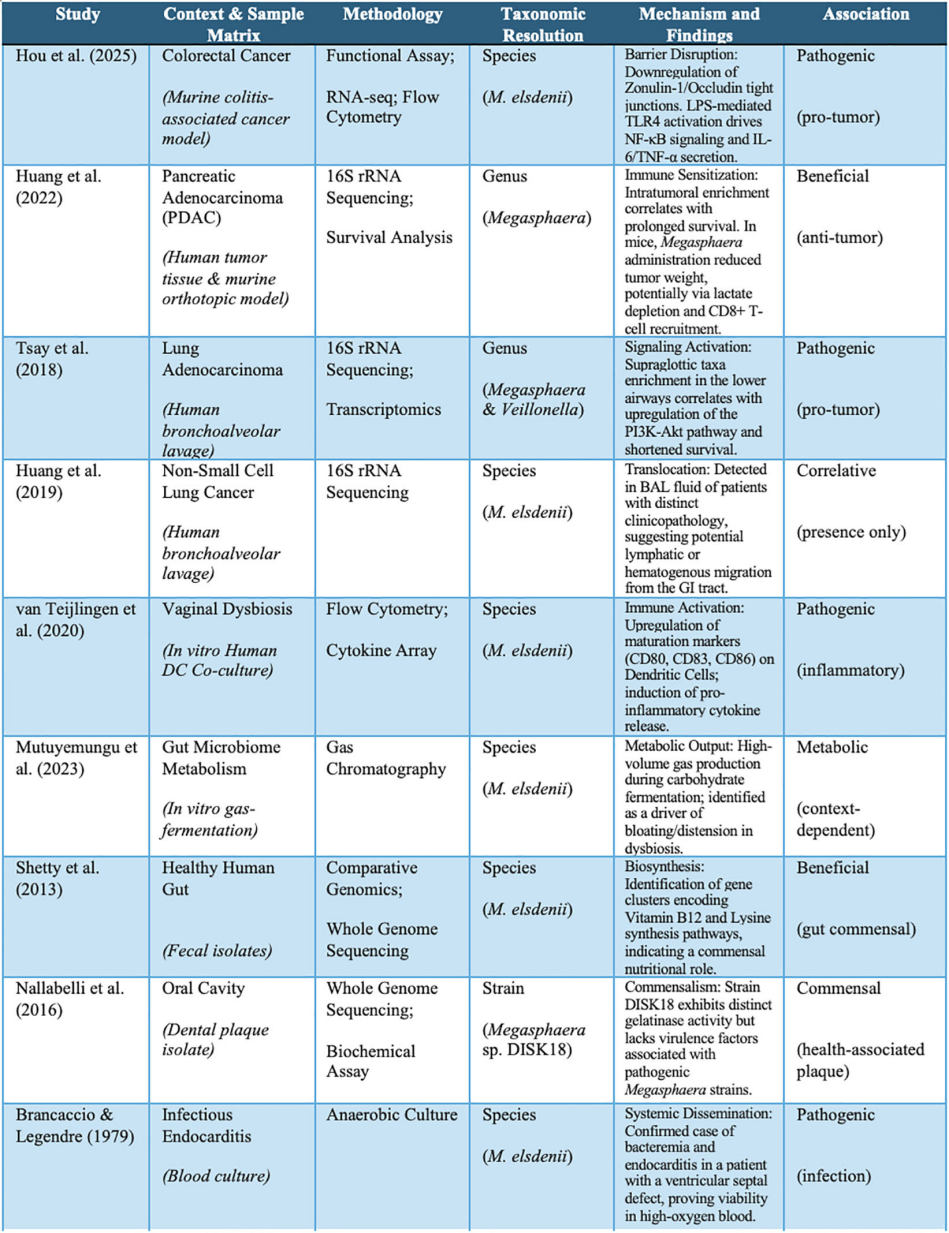
Summary of key studies examining the role of Megasphaera and related genera in cancer and human health. Studies are organized by clinical context, methodology, taxonomic resolution, proposed mechanisms, and association type.

Compared with better-studied taxa such as *Fusobacterium nucleatum*, *M. elsdenii* offers a compelling model of dualistic immunomodulation. While the species is largely implicated in epithelial barrier disruption and inflammation in the colon and vagina, recent evidence identifies *Megasphaera* sp. *XA511* as a distinct, therapeutically relevant gut-derived strain belonging to an as-yet unclassified *Megasphaera* species (not *M. elsdenii*) that defies this pathogenic paradigm by potentiating anti-tumor immunity in pancreatic models. These pathways are summarized schematically in **Figure 1**.

We focus on *M. elsdenii* as the prototypical gut microbiome species and propose that it functions not as a sole causal oncogenic driver but rather as a microbe that displays site-specific niche flexibility in a host of organs and tracts. While direct experimental evidence confirms its metabolic output of SCFAs and potential for dendritic cell activation, its systemic role remains partially inferred from parallel pathways observed in *Fusobacterium* and *Bacteroides* models. This review synthesizes these associative findings to generate testable hypotheses regarding the gut-lung axis.

## 2 Taxonomic and functional overview of *Megasphaera elsdenii*

*M. elsdenii* belongs to the class *Negativicutes*, order *Veillonellales*, and family *Veillonellaceae* (Campbell et al., 2015). Unlike typical gram-negative organisms, it possesses an atypical double-membrane structure within the Firmicutes lineage, conferring both Gram-negative staining and anaerobic resilience (Costerton et al., 1974; Marchandin et al., 2010; Antunes et al., 2016). The outer membrane contains lipopolysaccharides and a protein-rich S-layer that facilitates immune interaction and stability in low-oxygen conditions (Kojima and Kamio, 2012). Its peptidoglycan layer contains cadaverine cross-links and distinct D-glutamate residues that form amidation bridges critical for maintaining osmotic balance in acidic environments (Rands et al., 2019), making it particularly resilient in persisting across multiple mucosal environments. Beyond *M. elsdenii* the *Megasphaera* genus includes species adapted to distinct niches. *Megasphaera micronuciformis* has been reported in the human oral cavity with associations to periodontal disease in overabundant states, whereas *Megasphaera cervesiae* exemplifies environmental persistence through robust biofilm formation on brewery-associated surfaces (Nallabelli et al., 2016; Bittner et al., 2017). These examples highlight genus-wide niche diversity that parallels the metabolic flexibility described below.

Proteomic data suggest that *M. elsdenii* utilizes lysine- and glutamate-rich residues on its cell surface proteins, primarily its electron transfer flavoproteins that are critical for reduction-oxidation reactions, as a primary source of metabolism (Dwyer et al., 1999; Parker, 2003; Mohamed-Raseek and Miller, 2022). Other species of *Megasphaera* have a strong association with survival in biofilms (Bittner et al., 2017) which could contribute to its evasion of the immune system during periods of eubiosis.

*M. elsdenii* primarily metabolizes lactate through the acrylate and succinate pathways to produce short-chain fatty acids such as acetate, propionate, and butyrate (Prabhu et al., 2012), which regulate epithelial metabolism and immune tolerance (Mirzaei et al., 2021). It relies on several key enzymes, including lactate dehydrogenase, propionyl-CoA transferase, and butyryl-CoA dehydrogenase, to manage intracellular redox balance and characteristically prefers lactate as a carbon source over glucose (Marounek et al., 1989).

The resulting SCFAs interact with G-protein–coupled receptors (GPR41, 43, 109A) on epithelial and immune cells, modulating Treg differentiation and neutrophil chemotaxis, ultimately bolstering immune barrier function in intestinal epithelium through upregulation of occludins and inhibition of nuclear-factor kappa-beta (NF-κB) signaling as well as supporting mucosal immune tolerance (AnaChad et al., 2023).

## 3 Mechanistic pathways of microbial carcinogenesis

In homeostatic conditions, *M. elsdenii* participates in cross-feeding networks with butyrate producers, such as *Faecalibacterium prausnitzii*, to maintain epithelial energy balance (Louis and Flint, 2017). Under dysbiosis, however, the organism’s metabolic behavior shifts. Recent research by Hou et al. (2025) demonstrated that the overrepresentation of *M. elsdenii* in murine colonic models led to decreased expression of intestinal barrier proteins, including Zonulin-1, Zonulin-2, occludin, and claudin-2, increasing epithelial permeability, and activation of NF-κB-dependent, proinflammatory cytokines including IL-6 IL-1β and TNF-α (Hou et al., 2025). Further, RNA sequencing confirmed gut epithelial barrier dysfunction and tumorigenesis, namely through upregulation of CD11b+ dendritic cells, promoting a proinflammatory state in the colon (Hou et al., 2025). This study further reported that modulations in CD103b+ dendritic cells favored a pro-inflammatory state, through downstream expression of cytokines IFN-γ and IL-17a. This occurs primarily through activation of toll-like receptor 4 from lipopolysaccharide residues on the outer membrane of *M. elsdenii*; this increases expression of NF-κB and interferon regulatory factor 4 (IRF4). Although the role of IRF4 is controversial, a current paper found that elevated expression of IRF4 in immune cells was associated with decreased survival in a sizable cohort of patients with colorectal cancer (Tan et al., 2025).

Microbial involvement in carcinogenesis arises from three proposed major mechanisms: genotoxicity, chronic inflammation, and metabolic reprogramming (Schwabe and Jobin, 2013). In the case of *M. elsdenii*, inflammation and metabolic flux dominate. The overabundance of *M. elsdenii* increases local concentrations of propionate within the tumor microenvironment fueled primarily by lactate from the tumor itself. This further promotes hypoxia which theoretically promotes the stabilization of HIF-1α. Although direct evidence for *M. elsdenii-*derived HIF-1α stabilization *in vivo* is limited, analogous mechanisms in other lactate-producing anaerobes support this hypothesis (Kotlyarov, 2022). Simultaneously, TLR4 activation by its lipopolysaccharide-like outer membrane triggers NF-κB signaling, leading to elevated IL-17 and IL-23 production (Zhou et al., 2020).

This proinflammatory milieu may foster angiogenesis and epithelial proliferation through the PI3K–AKT–MAPK axis (Tsay et al., 2018; Jiang et al., 2019). Tsay et al. (2018) identified a *Megasphaera* signal enriched in the lower airways of lung cancer patients and associated with upregulated PI3K–Akt signaling; however, these 16S rRNA data provide only genus-level resolution and did not confirm *M. elsdenii* presence specifically. Within this limitation, we hypothesize that part of the pulmonary *Megasphaera* signal could reflect gut-derived immunogenic debris (such as OMVs) originating from the gastrointestinal tract, but this remains a non-causal, hypothesis-generating association. Lactate accumulation inhibits cytotoxic T-cell function and supports tumor-associated macrophage polarization toward the M2 phenotype (Fischer et al., 2007). These shifts parallel *Fusobacterium nucleatum*–associated colorectal cancer, though metabolic foundations differs (Castellarin et al., 2012). In *M. elsdenii* dysbiosis, the driver is more likely excessive lactate flux rather than direct adhesin-mediated invasion.

Studies have shown that serum IL-6 and IL-17 levels rise during intestinal dysbiosis and correlate with pulmonary Th17 activation (Bingula et al., 2017; Budden et al., 2017). This forms the conceptual basis for the gut–lung axis.

## 4 The gut–lung axis and dissemination of microbial signals

The gut–lung axis refers to the bidirectional communication between the intestinal and respiratory mucosa through microbial metabolites, immune mediators, and lymphatic trafficking (Huffnagle et al., 2017). Both organs share a common mucosal immune system in which lymphocytes primed in Peyer’s patches migrate through mesenteric lymph nodes and the thoracic duct to the pulmonary tissue (Bingula et al., 2017; Zhou et al., 2020).

The mesenteric lymph functions as an active conduit in which lipids, cytokines, danger-associated molecular patterns, and bacterial extracellular vesicles travel via the thoracic duct to the pulmonary circulation (Ma et al., 2021). Ryu et al. (2023) demonstrated that bacterial OMVs strongly induce macrophage pro-inflammatory activation and inflammatory lung responses via multiple signaling pathways, including TLR2, TLR4, and S100-A8-mediated DAMP signaling effects that exceed those of purified LPS alone. This migration transmits gut-imprinted cytokine profiles that influence local immunity in the lung, a process especially relevant in lung cancer (Hagihara et al., 2024).

Lymphatic dissemination allows microbial antigens to be transported by dendritic cells from the gut to distal sites (Li et al., 2024). Circulating metabolites such as butyrate and propionate can modulate alveolar macrophage activity and cytokine secretion. Experimental models show that antibiotic-induced depletion of gut microbes exacerbates airway inflammation, whereas recolonization with SCFA-producing bacteria restores epithelial balance (Mindt and DiGiandomenico, 2022). These findings support a model in which microbial metabolites, rather than viable bacteria, serve as the principal messengers in systemic immune modulation.

Both the gut and lung epithelia rely on pattern recognition receptors such as TLR2, TLR4, and NOD2 to maintain immune equilibrium. Persistent microbial stimulation upregulates NF-κB and AP-1 transcriptional programs, leading to chronic cytokine production and angiogenic signaling (Zhou et al., 2020). Elevated expression of IL-17, IL-23, and PD-L1 in both tissues suggests shared inflammatory pathways linking mucosal inflammation with tumor progression (Zhang et al., 2023).

Although *M. elsdenii* is non-motile and lacks flagellar or spore-forming capacity, several immunologic and lymphatic routes could enable its molecular or cellular signatures to influence pulmonary tissues. Tsay et al. (2018) documented enrichment of *Veillonella* and *Megasphaera* genera in bronchoalveolar lavage samples of lung adenocarcinoma and correlated this with increased expression of PI3K-Akt signaling. In Peyer’s patches, dendritic cells process *M. elsdenii* antigens and prime lymphocytes that enter mesenteric lymphatics and drain through the thoracic duct into systemic circulation, where the lung represents one of the first microvascular beds exposed to lymph-borne immune mediators (Nguyen et al., 2020; Ma et al., 2021). Mesenteric lymph is a conduit for both soluble factors and vesicular material; bacterial outer-membrane vesicles and exosomes enriched in lipopolysaccharide, nucleic acids, and metabolic enzymes can survive anoxic transport and interact with pulmonary endothelial and myeloid cells upon arrival (Guo et al., 2023). These vesicles can activate endothelial TLR4 and NLRP3 inflammasome pathways, increasing vascular permeability and facilitating deposition of microbial products without viable bacterial migration. We therefore propose, as a testable hypothesis, that Megasphaera-derived OMVs or related vesicles could enter mesenteric lymph, transit the thoracic duct, and deliver immunogenic cargo to the lung microvasculature. This aligns with evidence that OMVs bypass epithelial barriers and activate dendritic cells and macrophages via (CD40, CD80, CD86) at distal sites (Jalalifar et al., 2023).

Despite its non-motility, *Megasphaera* species have been found in the bronchoalveolar lavage fluid of lung cancer patients (Huang et al., 2019). These observations are likewise based on 16S rRNA amplicon profiling, which cannot distinguish viable from non-viable bacteria, nor resolve species within the *Megasphaera* genus, and therefore do not establish a causal role for *M. elsdenii* in lung cancer. However, this paper proposes a modified hypothesis wherein detected *M. elsdenii* DNA in the lung may represent translocated Outer Membrane Vesicles (OMVs) or cell wall fragments rather than active infection. These fragments are sufficient to trigger TLR4-mediated inflammation in the alveolar niche without requiring the metabolic activity of live bacteria.

## 5 Microbial metabolites and the tumor microenvironment

**Figure 2:**
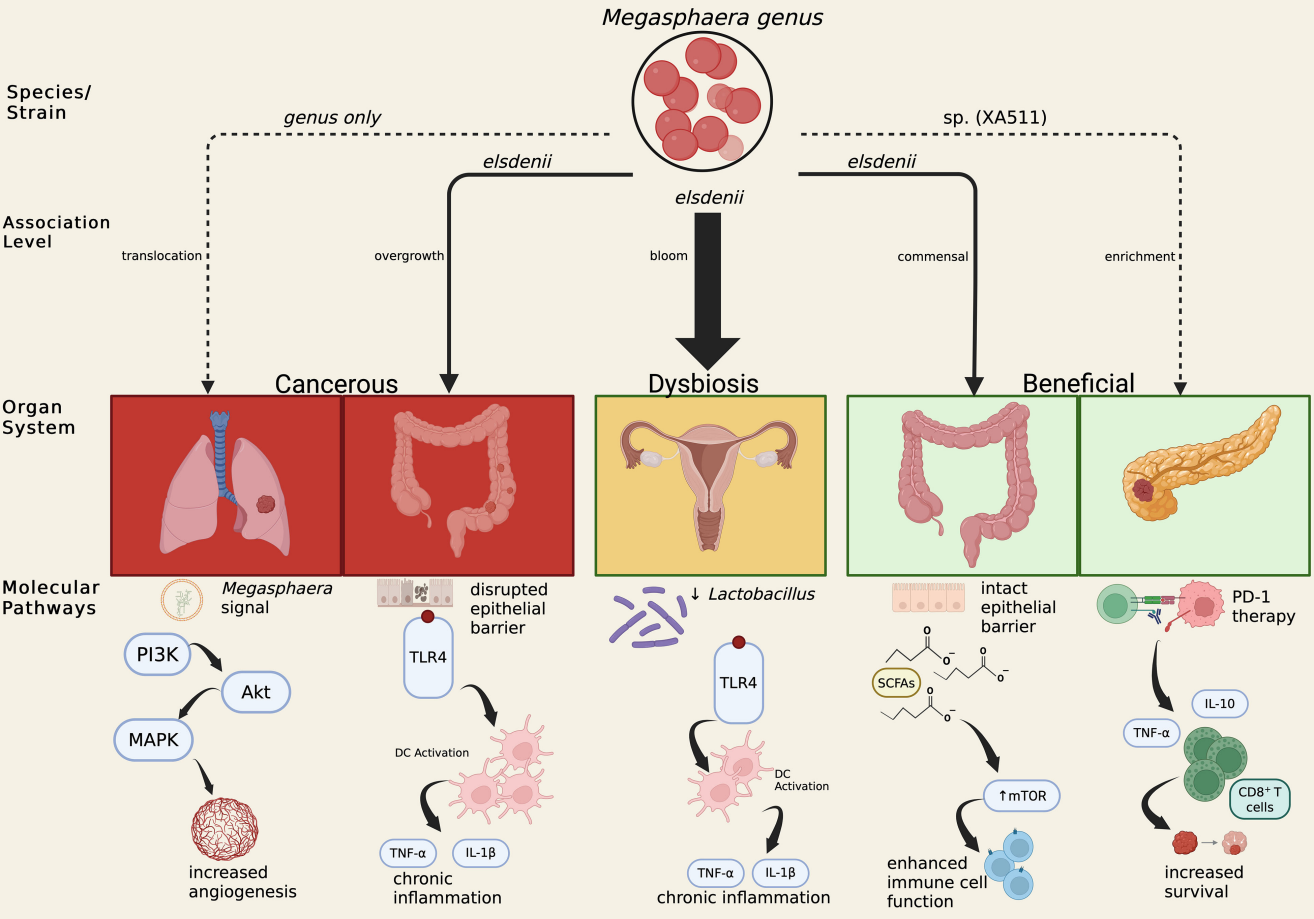
Schematic overview of the context-dependent roles of the Megasphaera genus across anatomical sites. Pathways include gut–lung axis signaling, colonic barrier disruption, vaginal dysbiosis, and protective immunomodulation in pancreatic tumor microenvironments.

In the colon, *M. elsdenii* overabundance correlates with proinflammatory cascades and barrier loss. This pathogenic profile stands in sharp contrast to the specific isolate *Megasphaera* sp. XA511. In the context of pancreatic ductal adenocarcinoma (PDAC), Huang et al. (2022) identified that *Megasphaera* sp. XA511 functions as a protective immunomodulator. This was further positively associated with survival in Chinese PDAC patients. To further test the systemic immunomodulatory potential of these gut-derived bacteria, the authors utilized a murine 4T1 breast cancer model which was chosen for its aggressive immunogenic phenotype. They found that oral administration of *Megasphaera* sp. *XA511* significantly enhanced anti-PD-1 efficacy. While this model relies on breast cancer cell lines, the findings suggest that *Megasphaera* may exert effects via systemic immune sensitization rather than requiring direct colonization of the pancreatic duct. The same metabolic flexibility that drives dysbiosis-associated inflammation may, under nutrient-limited tumor microenvironments, restore redox equilibrium and sensitize tumors to immune checkpoint blockade (Li et al., 2024). **Figure 2** summarizes these findings.

Microbial metabolites function as systemic messengers that reprogram host cell metabolism and immunity. At physiologic levels, SCFAs promote epithelial repair and immune tolerance by enhancing histone acetylation and Treg expansion (Maslowski et al., 2009). However, during *M. elsdenii* overgrowth, excessive lactate and propionate accumulation alters redox potential and may stabilize HIF-1α, stimulating angiogenic and glycolytic pathways (Fischer et al., 2007; Hou et al., 2025), which could result in metabolic rewiring promoting resistance to apoptosis and immune surveillance.

The effect of these metabolites varies by tissue context. In the colon and lung, high concentrations of SCFAs enhance inflammatory signaling and oxidative stress. In contrast, in pancreatic tumors, which are known for their high heterogeneity and plasticity of fibroblasts (Kung et al., 2025), lactate utilization by *Megasphaera* strains such as sp. XA511 may attenuate acidosis. Such environment-dependent character aligns with the broader principle that the metabolic environment, often varying between cancer types and location, determines whether microbial byproducts act as carcinogens or therapeutic allies (Pérez-Tomás and Pérez-Guillén, 2020).

## 6 Discussion

This mini-review examines *M. elsdenii* as a gut microbe in cancer and outlines future clinical and experimental directions. In the lung, existing data rely solely on 16S rRNA surveys that detect *Megasphaera* at the genus level, so any proposed contribution of *M. elsdenii* to pulmonary tumorigenesis should be regarded as speculative. To move beyond a binary classification of commensal versus pathogen, we propose that the immunometabolic impact of the genus is determined by both anatomical niche and strain specificity. While *M. elsdenii* acts as a consistent driver of inflammation in the vaginal and colonic compartments, *Megasphaera* sp. XA511 exemplifies a ‘metabolic specialist’ capable of reversing immunosuppression in the pancreatic microenvironment by altering Warburg effect dynamics. Future therapeutic strategies must therefore distinguish between broad-spectrum antibiotic suppression of *M. elsdenii* in lung/gut dysbiosis versus the precision administration of *XA511* as an adjuvant for immunotherapy.

In hypoxic tumor microenvironments like PDAC with high lactate burdens, *Megasphaera* sp. *XA511* likely acts protectively by metabolizing excess lactate into butyrate, thereby reducing local acidosis and enhancing CD8+ T-cell function. Second is barrier integrity. In the presence of compromised colonic tight junctions such as in colitis, *M. elsdenii* is predicted to drive tumorigenesis via LPS translocation and TLR4 activation. Third is the anatomical niche. While SCFAs are tolerogenic in the gut, their accumulation in the sterile lung environment is often pro-inflammatory and potentially drives Th17 polarization in lung adenocarcinoma. *M. elsdenii* detection in the lung is far less consistent than other compartments reinforcing the hypothesis that pulmonary presence represents transient translocation rather than stable resident colonization. The most reproducible human signal of *M. elsdenii* remains colonic and vaginal inflammatory association, whereas pulmonary detection is limited and inconsistent.

To rigorously test whether *M. elsdenii* is a driver of lung tumorigenesis and not merely a biomarker, future studies must employ gnotobiotic murine models colonized specifically with *M. elsdenii*. The central hypothesis would be falsified if antibiotic clearance of *M. elsdenii* from the gut fails to attenuate pulmonary inflammation, or if administration of sterile bacterial filtrates fails to recapitulate the pro-tumorigenic phenotype observed with live inoculation. Integrating metabolomics and immunophenotyping in longitudinal human cohorts will help clarify temporal relationships between dysbiosis and cancer initiation.

Restoring microbial equilibrium could attenuate cancer-promoting inflammation. Strategies include dietary modulation to enhance butyrate producers, prebiotic supplementation to reduce Veillonellaceae expansion, and pharmacologic targeting of GPR41/43 or TLR4 signaling (Tsvetikova and Koshel, 2020). In PDAC, *M. elsdenii*’s metabolic activity may be harnessed to normalize lactate flux and improve immunotherapy outcomes (Huang et al., 2022). Conversely, in colorectal and pulmonary inflammatory states, targeted suppression or rebalancing of *Megasphaera* populations may prove beneficial (Tsay et al., 2018; Hou et al., 2025). The capacity to preferentially utilize lactate, generate diverse SCFAs, and synthesize essential nutrients such as lysine and B vitamins likely underlies the ability of the *Megasphaera* genus to occupy multiple anatomical niches and shift between commensal and proinflammatory roles.

Viewing *M. elsdenii* as a context-dependent modulator rather than a unidirectional pathogen reframes its role in cancer biology and our understanding of beneficial microbes. Redefining the molecular thresholds that separate its commensal from pathogenic behavior, can allow future projects to explore whether modifying its abundance or activity can influence therapeutic response. Specifically, bacteriophage-based strategies targeting *Megasphaera* could offer strain-level control without the collateral damage of broad-spectrum antibiotics, although *Megasphaera*-specific phages have not yet been characterized in oncologic settings. Patients with *Megasphaera*-enriched tumors may serve as candidates for precise microbial manipulation, consistent with a personalized medicine approach. Such insights may ultimately transform microbial management from supportive care into a cornerstone of oncologic prevention and treatment.

## Glossary

*M. elsdenii*: *Megasphaera elsdenii*
LPS: Lipopolysaccharide
OMV(s): Outer Membrane Vesicle(s)
CD40/CD80/CD83/CD86: Cluster of Differentiation
DC(s): Dendritic Cell(s)
Treg(s): Regulatory T Cell(s)
Th1/Th17: T Helper Type 1/Type 17 Cell(s)
B cell(s): B Lymphocyte(s)
SCFA(s): Short-Chain Fatty Acid(s)
NF-κB: Nuclear Factor Kappa-light- chain-enhancer of Activated B Cells
mTOR: Kinase Integrating Nutrient and Growth Signals
TLR2/TLR4: Toll**-**Like Receptor 2/4
NOD2: Nucleotide-binding Oligomerization Domain-containing Protein 2
NLRP3: NOD-like Receptor Family Pyrin Domain-containing 3 (Inflammasome)
HIF-1α: Hypoxia-Inducible Factor 1-Alpha
PI3K: Phosphoinositide 3-Kinase
AKT: Protein Kinase B
MAPK: Mitogen-Activated Protein Kinase
PD-L1: Programmed Death-Ligand 1
PD-1: Programmed Cell Death Protein 1
AP-1: Activator Protein 1
Interleukins: IL-1β: Interleukin 1 Beta
IL-6: Interleukin 6
IL-17a: Interleukin 17a
IL-23: Interleukin 23
TNF-α: Tumor Necrosis Factor Alpha
IFN-γ: Interferon Gamma
IRF4: Interferon Regulatory Factor 4
CCL25/CCL28/CXCL16: Chemokine Ligands involved in lymphocyte trafficking
CCR9: C–C Chemokine Receptor 9
α4β7: Integrin Alpha-4 Beta-7
Zonulin-1/Zonulin-2: Tight Junction Regulatory Proteins
Claudin-2: Tight Junction Protein
Occludin: Tight Junction Protein
PDAC: Pancreatic Ductal Adenocarcinoma
RNA-seq: RNA Sequencing
ROS: Reactive Oxygen Species.
